# Near-Infrared Spectroscopy Used to Assess Physiological Muscle Adaptations in Exercise Clinical Trials: A Systematic Review

**DOI:** 10.3390/biology11071073

**Published:** 2022-07-19

**Authors:** Marcelo Tuesta, Rodrigo Yáñez-Sepúlveda, Humberto Verdugo-Marchese, Cristián Mateluna, Ildefonso Alvear-Ordenes

**Affiliations:** 1 Exercise and Rehabilitation Sciences Laboratory, School of Physical Therapy, Faculty of Rehabilitation Sciences, Universidad Andres Bello, Santiago 7591538, Chile; marcelo.tuesta@unab.cl; 2 Laboratory of Sport Sciences, Centro de Medicina Deportiva Sports MD, Viña del Mar 2521156, Chile; drhumbertoverdugo@gmail.com; 3 Applied Physiology Laboratory (FISAP), Institute of Biomedicine (IBIOMED), University of León, 24071 León, Spain; rodrigo.yanez@uvm.cl; 4 School of Education, Pedagogy in Physical Education, Universidad Viña del Mar, Viña del Mar 2572007, Chile; 5 Physical Education School, Pontificia Universidad Católica de Valparaíso, Valparaíso 2530388, Chile; matelunanc@gmail.com

**Keywords:** NIRS, physical exercise, hemoglobin, muscle oximetry

## Abstract

**Simple Summary:**

In recent years, physical exercise has been used as a therapeutic strategy in various clinical conditions, with pleiotropic benefits. Near-infrared spectroscopy (NIRS) has been positioned as a tool to analyze effects on muscle oxygenation, also allowing knowledge of adaptations on microvascular levels and muscle metabolism in subjects with central and peripheral vascular alterations, as well as cardiovascular, metabolic, and/or musculoskeletal diseases. This knowledge can help to guide therapeutic exercise specialists in decision making regarding the prescription and follow up of physical exercise as a therapeutic tool in the observation of acute or chronic adaptations to improve efficiency in the treatment and recovery of these patients. This review presents an overview of the effects of exercise clinical trials on muscle oxygenation in different pathologies and the technical characteristics related to the equipment used.

**Abstract:**

Using muscle oxygenation to evaluate the therapeutic effects of physical exercise in pathologies through near-infrared spectroscopy (NIRS) is of great interest. The aim of this review was to highlight the use of muscle oxygenation in exercise interventions in clinical trials and to present the technological characteristics related to the equipment used in these studies. PubMed, WOS, and Scopus databases were reviewed up to December 2021. Scientific articles that evaluated muscle oxygenation after exercise interventions in the sick adult population were selected. The PEDro scale was used to analyze the risk of bias (internal validity). The results were presented grouped in tables considering the risk of bias scores, characteristics of the devices, and the effects of exercise on muscle oxygenation. All the stages were carried out using preferred reporting items for systematic reviews and meta-analyses (PRISMA). The search strategy yielded 820 clinical studies, of which 18 met the eligibility criteria. This review detailed the characteristics of 11 NIRS devices used in clinical trials that used physical exercise as an intervention. The use of this technology made it possible to observe changes in muscle oxygenation/deoxygenation parameters such as tissue saturation, oxyhemoglobin, total hemoglobin, and deoxyhemoglobin in clinical trials of patients with chronic disease. It was concluded that NIRS is a non-invasive method that can be used in clinical studies to detect the effects of physical exercise training on muscle oxygenation, hemodynamics, and metabolism. It will be necessary to unify criteria such as the measurement site, frequency, wavelength, and variables for analysis. This will make it possible to compare different models of exercise/training in terms of time, intensity, frequency, and type to obtain more precise conclusions about their benefits for patients.

## 1. Introduction

Oxygen is a fundamental molecule for maintaining energy production in tissues. During exercise, a higher muscle metabolism increases the demand for this chemical element, which must be rapidly delivered to the mitochondria for energy production. In healthy subjects, the level of oxygen in the mitochondria is the result of a dynamic balance between blood transport capacity and tissue uptake [1]. Therefore, an increase in the flow of oxygenated blood to the active musculature during exercise ensures, in part, the maintenance of the energy production necessary for the effort. Thus, to know the level of muscle oxygenation in situ allows the identification of oxidative metabolism. In recent years, the measurement of muscle oxygenation through near-infrared spectroscopy (NIRS) has been of great interest [2,3]. NIRS is a non-invasive, optical method that uses an infrared light beam to pass through biological tissues [4]. This technology has been used to detect real-time changes in oxygenation in tissues, mainly brain and muscle tissues, with a high level of reliability [3,4,5,6,7]. Once in the muscle, the infrared light emanating from the device is absorbed by the chromophores of primary interest (e.g., total hemoglobin (tHb), deoxyhemoglobin (HHb), oxyhemoglobin (O_2_Hb)) and reflected by the rest of the tissue [8,9], causing an attenuation of the intensity of the infrared beam emerging from the NIRS [10]. 

There are four main technological techniques used by NIRS equipment in muscle oxygenation studies in humans. Continuous wave (CW) is very popular due to its versatility and low cost; however, its main limitation is that it only detects relative changes of chromophores in arbitrary units or percentage changes in their concentrations with respect to the baseline set as zero using the modified Beer–Lambert law [11]. More sophisticated techniques are frequency domain (FD), time domain (TD), and spatially resolved or multiple distances (SRS), the main advantage of which over CW-NIRS is that they can measure the absolute concentrations of chromophores (i.e., O_2_Hb and HHb) present in tissues. Currently, the simultaneous integration of the modified Beer–Lambert law [11] and the SRS (multi-distance) configuration/method in CW-NIRS equipment makes it possible to obtain the changes in the micromolar O_2_Hb and HHb concentrations and, with these, the tissue oxygen saturation (in %). The latter reflects the dynamic balance between oxygen delivery and utilization in the tissue, i.e., the skeletal muscle fractional oxygen extraction [3]. Briefly, SRS is a method that measures the attenuation of light using different wavelengths at multiple distances from the emitted light [12].

Modifications in the concentrations of NIRS variables have been used to interpret some physiological adaptations of muscle tissue. For example, the measurement of differential hemoglobin ([O_2_Hb] − [HHb]) during a protocol of arterial occlusion of an extremity (e.g., ~5 min) has been used to determine the oxygen consumption of distal musculature. Additionally, total hemoglobin ([O_2_Hb] + [HHb]) during reperfusion (post occlusion) can be interpreted as changes in blood volume [3]. To analyze oxidative metabolism, the study of oxygen saturation during reperfusion is recommended [13]. These changes are associated with functional parameters in patients and have received increased interest in different areas of study [14,15,16,17], such as sports training [2,4,18,19,20,21,22,23,24]. The use of NIRS has also even been validated in underwater exercise [25] and rehabilitation [26,27,28,29]. Some studies used NIRS to monitor circulatory adaptation (tissue perfusion) and muscle oxygenation following clinical trials of therapeutic exercise [30,31,32,33,34,35,36], as well as during sports training [18,37].

A recent review demonstrated that NIRS has remarkable potential for use in the assessment of acute and chronic exercise response [18]. However, it is still necessary to discuss the adaptive responses to exercise in clinical situations. In this sense, NIRS has the potential to elucidate the responsibility of the central and/or peripheral component in the limitation of pathology-induced exercise tolerance. At the central level, the failure of cardiac pump function during exertion limits the convective capacity of oxygenated blood to reach the active locomotor musculature, as occurs in patients with heart failure. On the other hand, at the peripheral level, the detrimental effect of diabetes mellitus on the muscular metabolic capacity for energy production (metabolic inflexibility) is recognized, as well as atherosclerosis (peripheral artery disease), which limits blood perfusion to the active locomotor musculature, affecting the adequate delivery of oxygen [38,39]. This allows training methods (types: continuous or interval training) and other parameters (frequency, intensity, duration) to be evaluated to discern which elicits the greatest associated health benefits.

The aim of this review was to highlight the use of muscle oxygenation in exercise interventions in clinical trials and to present the technological characteristics related to the equipment used in these studies. Among the results of the analysis, we present the methodological quality through the PEDro scale, provide information on the technical characteristics of measurement of the NIRS devices used, and, finally, highlight the results relating to muscle oxygenation, blood flow, or metabolism in clinical trials that used physical exercise training as therapy.

## 2. Materials and Methods

The present study was conducted according to the recommendations of preferred reporting items for systematic reviews and meta-analyses (PRISMA) [40]. The study is also registered in the International Prospective Register of Systematic Reviews (PROSPERO) (identifier CRD42020220997). 

### 2.1. Scientific Literature Search

An electronic search for clinical trial studies was performed in the PubMed, WOS, and Scopus databases for material published before December 2021. For this purpose, the following MeSH (medical subject headings) terms were used together: “Near-infrared spectroscopy” and “exercise”, which were joined using the Boolean method “AND”. In addition, the complementary words excluded from MeSH, “exercise therapy”, “physical exertion”, “physical fitness”, “sports”, and “exercise movement techniques”, were included in the search, also joined by the “AND” connector, and, finally, the Boolean “OR” was used to join the searches. We also reviewed the reference lists of the retrieved studies as an additional process for determining possible studies to include in this review. As a precondition, all studies had to have been conducted in adults with a disease.

### 2.2. Eligibility Criteria

The population, intervention, comparison, outcome, and study design (PICOS) approach was applied to systematically define the eligibility criteria. The assigned inclusion criteria were: i. Manuscript written only in the English language; ii. Only articles published in peer-reviewed journals; iii. Adult and elderly population (>18 years); iv. Female and/or male; v. Muscle tissue oxygenation analysis with commercial NIRS using the Beer–Lambert law to detect chromophores; vi. Clinical trials with control group or placebo; vii. Interventions with exercise in the experimental group (e.g., aerobic, strength, water sports, combinations, and others). Exclusion criteria were studies that included: i. Use of ergogenic aids; ii. Other type of intervention in the experimental and/or control group and/or placebo (e.g., cold water immersion, electrostimulation, hypoxia, vibration, occlusion, oxygenation); iii. Articles that did not detail the characteristics of the NIRS used; iv. Lack of information on the frequency, intensity, duration, or type of physical exercise intervention; and v. Articles without control group and/or placebo.

### 2.3. Data Extraction and Quality Assessment

Based on the eligibility criteria, two investigators (R.Y. and C.M.) independently reviewed and selected the articles. In case of differences between the two, a third researcher (M.T.) acted as mediator for the inclusion or exclusion of the studies in the review. The data obtained from these articles were: first surname of the first author, year of publication, name of the NIRS device, manufacturer and country of origin, technical characteristics, objective of the study, methodology of the intervention, anatomical site of device placement, and the results obtained relating to the outcome of muscle oxygenation in the studies used; significant effects were considered to have a *p*-value < 0.05. The Physiotherapy Evidence Database (PEDro) scale was used to assess the risk of bias in the studies, as it is valid for evaluating the methodological quality of clinical trials [41]. The PEDro scale is designed with 11 items, all but 1 of which (external validity) give a point if present, so the final score should be in the range between 0 and 10 points. According to the predetermined cut-off points, the studies are classified as high (8 to 10), medium (4 to 7), or low (less than 4) quality [42]. The assessment was performed independently by two researchers (R.Y. and C.M.).

## 3. Results and Discussion

### 3.1. Description of the Selected Studies

**Figure 1:**
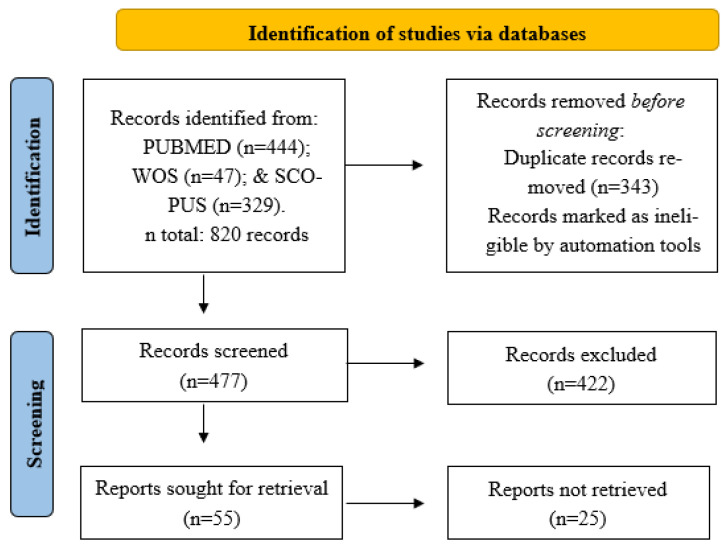

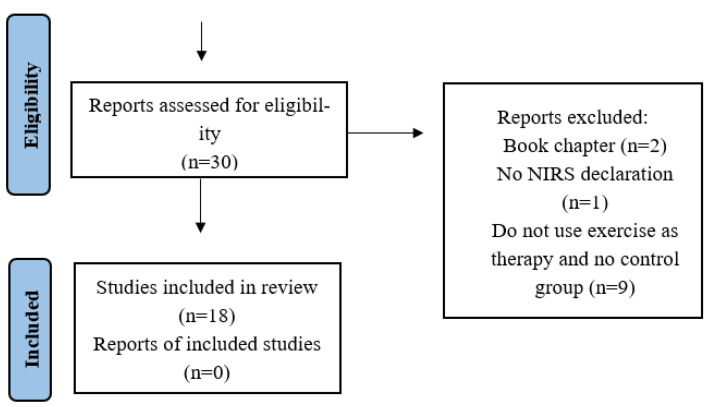
Flow diagram (PRISMA) of the item selection process.

Figure 1 summarizes the search and selection procedures of the scientific articles according to the PRISMA model. A total of 820 scientific articles were collected using the keywords in the MEDLINE/PubMed (*n* = 444), WOS (*n* = 47), and Scopus (*n* = 329) databases (Appendix A, Table A1). Subsequently, 343 duplicate articles were excluded, resulting in a total of 477 remaining articles. Of the total number of scientific articles, 422 were eliminated after reading the titles and abstracts for dealing with topics unrelated to physical exercise interventions, leaving 55 studies for full reading. In the first stage, 25 articles that included other interventions in addition to exercise were eliminated, leaving 30 articles for complete reading and review of the manuscript. Finally, based on the eligibility criteria, 12 studies were eliminated, leaving a total of 18 articles for review. The cross-checking of the references of the articles obtained from the bibliographic databases did not yield any new results.

### 3.2. Methodological Quality Assessment

Table 1 shows the quality results of the 18 selected studies according to the PEDro scale. All studies were classified as medium quality [30,31,43,44,45,46,47,48,49,50,51,52,53,54,55,56,57,58]. The lowest score was the Beckitt et al. [43] study, with 4/10 points, while the highest was that of Gardner et al. [47], Gildea et al. [48], Mezzani et al. [52], and Tew et al. [58], with 7/10 points each. The aspects that were not developed in any of the studies were “all subjects were masked” and “all therapists who administered therapy were masked”. These biases are frequently observed in literature reviews of clinical trials involving patients. The nature of the intervention (non-pharmacological), the clinical ethical context of the information that the patient should receive about his or her treatment, and the need for health specialists in the care, follow up, and evaluation of the patient, among other things, limit recommended actions, such as no contact between the placebo and experimental groups or lack of awareness of the existence of the placebo, the nature of the placebo, or the hypothesis of the trial (which is control and/or experimental). In the case of “unblinded” therapists, it is recommended that they are not in contact with the experimental group. It is recommended that they should not be in charge of other care or objective measurements [59]. In this sense, if we discard both unattainable aspects for the studies included in this review, the maximum possible score that could be obtained is 8 points out of 10. As it is recognized, the PEDro scale recommends that interventions with physical exercise present a total score of at least 8 points out of 10 [60]. In this review, no study complied with this recommendation (Table 1). Therefore, further efforts are necessary to improve quality indicators to avoid increasing the risk of bias in future studies. For the time being, the conclusions should be viewed with caution.

Items in the PEDro scale: 1: eligibility criteria were specified; 2: subjects were randomly assigned to groups; 3: assignment was masked; 4: groups were similar at baseline in relation to the most important prognostic indicators; 5: all subjects were masked; 6: all therapists who administered therapy were masked; 7: all assessors who measured at least one key outcome were masked; 8: measures of at least one of the key outcomes were obtained from more than 85% of the subjects initially assigned to groups; 9: results were presented for all subjects who received treatment or were assigned to the control group or, when this could not be achieved, data for at least one key outcome were analyzed by “intention to treat”; 10: results of statistical comparisons between groups were reported for at least one key outcome; and 11: the study provided point measures and variability for at least one key outcome. Item 1 was not taken into account for the final score.

### 3.3. Characteristics of the NIRS Devices in Exercise Clinical Trials

An increase in the use of NIRS equipment to analyze muscle oxygenation with physical exercise as an intervention was observed. Some characteristics related to its use that favor this increase are its non-invasiveness, portability, real-time measurement, that it does not require trained personnel, its adequate measurement depth, versatility in the measurement of different muscle groups [12], and, in the most current equipment, the direct determination of molar concentrations of the chromophores present in the tissue (non-arbitrary units), as recently validated [61]. Table 2 shows that 11 models of NIRS device, belonging to nine manufacturers, were used in 18 clinical trials that evaluated the effects of physical exercise on tissue oxygenation in subjects with pathologies.

According to the NIRS techniques of the equipment used in the clinical exercise studies reviewed, the majority (9 of 11) used CW, of which eight included a multi-distance analysis (SRS) and only one was single distance. The remaining NIRS teams (2 of 11) used frequency domain. Precisely, these last-mentioned devices (DCS FD-NIRS and Oxyplet TS) could measure the O_2_Hb and HHb in absolute quantitative units in micromoles (μmol), and, thus, the tHb and DiffHb. Changes in the concentrations of the same variables were measured by the Portamon, Oxymon MkIII, NIRsrs, NIRO-300, NIRO-200, and BOM-L1TR. The CW-NIRS (NIM Inc., Philadelphia, PA, USA) and HEO-100 could measure these changes in percentages or arbitrary units from the baseline, respectively. The Inspectra Spectrometer 325 NIRS could obtain only tissue saturation. Regarding tissue saturation, HEO-100 was the only NIRS which used a CW single-distance technique that did not measure tissue saturation (Table 2). The tissue saturation level (SpO_2_) is a widely used parameter to measure oxygenation; others, such as HHb, allow the study of muscle oxygen consumption (mVO_2_) [62], and the tHb of blood flow changes in the muscle [63] through estimation. tHb is of vital importance when the aim is to study oxygenation adaptations in patients with compromised peripheral blood distribution, as occurs in some pathologies such as peripheral artery disease (PAD).

Regarding wavelength, only four had four different wavelength bands [30,31,43,45,46,47,51,53,55,56,58], three devices had three wavelengths [44,48,50], and four had only two [49,52,54,57]. The shortest wavelength observed was 680 nm in the Inspectra Spectrometer 325 manufactured by Hutchinson Technology Inc., and the longest was 905 nm in the NIRO-300 and Oxymon Mk III of the manufacturers Hamamatsu Photonics K.K. and Artinis Medical Systems, respectively (Table 2). This range in the near-infrared spectrum was the most suitable for use in the measurement of muscle oxygenation with NIRS in muscles due to the low absorption of this tissue [64].

### 3.4. Adaptations in the Oxygenation, Blood Flow, and Muscle Metabolism in Exercise Clinical Trials

Table 3 summarizes the research methodological characteristics and results, that is, oxygenation, perfusion, and metabolism muscle changes in the clinical studies that used NIRS. Here, we can see that the patients who participated in the studies (*n* = 861) were suffering from PAD (*n* = 527) [30,43,44,45,47,53,58], type 2 diabetes mellitus (T2D; *n* = 28) [48], heart failure (HFrEF; *n* = 99) [46,49,52], chronic kidney disease (CKD; *n* = 68) [31,50], anterior cruciate ligament reconstruction (ACLR; *n* = 24) [54], mitochondrial and McArdle’s muscle diseases (MMD; *n* = 13) [55], chronic myalgia (CM; *n* = 39) [56], acute myocardial infarction (AMI; *n* = 16) [57], and multiple sclerosis (MS; *n* = 46) [51].

#### 3.4.1. Peripheral Artery Disease

In clinical trials of subjects with PAD, aerobic exercises, such as a walking program, are traditionally used [44,45]. Briefly, a traditional walking protocol (TWP) consists of walking close to the painful threshold for as long as possible according to tolerance. A progression of intensity, and especially of exercise duration at each session, is expected. The sudden intolerance to effort imposed by the limitation to the increase in blood flow required by active musculature during physical activity is controlled with some efficacy. Recovery intervals begin with painful claudication and end with the cessation of symptoms. In this review, most studies with TWP protocols in patients with PAD showed an increase in exertion tolerance [30,44,45] in conjunction with increased blood flow [30,44], oxygen consumption [30,43,44] and delivery [44], and muscle desaturation during exercise, measured by NIRS [30,44,53]. These adaptations were related to a greater tolerance to effort, especially in terms of elapsed time in walking tests. Using 60 min of treadmill walking intervals at 2 mph with an increasing gradient (2% per 2 min) until claudication, the Baker et al. [44] group observed an increase in walking time, blood flow, and oxygen desaturation level during an exercise at maximal intensity in PAD patients’ gastrocnemius muscles. Previously, Manfredini et al. [30] observed that an unstructured walking training protocol, with a recommendation to maintain conditions like the structured one (six sessions of 20–30 min per week), did not improve the resting muscle oxygen consumption, calculated by analyzing the rate of increase in HHb during venous occlusion in the resting supine position [65]. A lack of exercise structuring may have resulted in an ischemic effect unable to trigger hemodynamic adaptive responses, such as collateral vascularization and endothelial function, as well as increased oxidative and glycolytic anaerobic metabolism, limiting the increase in the individual’s functional capacity. However, the structured protocol used in the Manfredini study [30] improved the muscle oxygenated hemoglobin levels (in arbitrary units) in the medial gastrocnemius and perfusion according to the ankle-brachial index during an incremental exercise test, along with an increased distance to claudication (two sessions of 10 min of walking at 20–30% below pain threshold speed). Notably, this was the only study [30] that had a long-term intervention (8.5 months), as all others used a 3-month intervention [43,44,45,47,53,58]. However, when TWP was performed, along with the use of canes, a smaller decrease in medial gastrocnemius hemoglobin saturation was observed during a treadmill exercise test compared to in the group that did not use canes [45]. It is possible that the weight unloading caused by the canes decreased the muscular work of the lower limbs, limiting the metabolic demand and using oxygen imposed by the active musculature. On the other hand, Monteiro et al. [53] increased muscle work by using ankle weights (intensity). During 3 months of training with three sessions per week (ses/wk), these patients carried out 15 min of walking on the floor with ankle weights (increasing progressively from 0.5 to 2 kg) or 30 min of walking on the floor and 30 min on treadmill at floor walking speed without inclination (an increase of 0.2 km·h^−1^ with the cessation of symptoms). Both groups decreased the rate of muscle desaturation in the medial gastrocnemius during exercise, but the group without ankle weights experienced greater changes. In addition, the level of muscle saturation during an exercise test remained higher in the conventional walking group (without ankle weights), despite the level of muscle ischemia. This result was also associated with an increase in exercise time during the test in this group but not in those using ankle weights. The changes may have depended on a longer time of hypoxic stimulus during the sessions, which was limited by the increase in intensity. This demonstrated that the TWP provokes a greater distribution and utilization of oxygen by the active musculature in PAD, improving exercise tolerance. Using NIRS, the study by Beckitt et al. [43] demonstrated that a group of patients with PAD who underwent angioplasty experienced significantly increased muscle fractional oxygen extraction (lower hemoglobin desaturation) in the lateral gastrocnemius during a submaximal test, but the exercise-only group (10′ warm up, five-station circuit, 8′ each station) did not. It should be noted that this last-mentioned study, unlike in other studies in PAD, used a smaller number of weekly sessions than the rest (only 2 ses/wk). However, both groups (angioplasty versus exercise) alone improved muscle reoxygenation during recovery after arterial occlusion. It was seen from muscle saturation level at the mean half-time recovery point that it had returned to its pre-exercise resting value. Although NIRS was able to differentiate the benefits of both interventions in patients with PAD, their association with hemodynamic and/or metabolic physiological changes still needs to be determined. With this, the use of NIRS in PAD allows guidance and control of interventions such as angioplasty, physical exercise, and/or modification of the use of pharmacology for meeting the patient’s clinical goals. 

Tew et al. [58] observed the effects of arm-crank exercise training on lower-limb O_2_ delivery in patients with intermittent claudication by PAD. Here, an increase in the time to reach a minimum of oxygen muscle saturation in the gastrocnemius at maximal and submaximal intensity during an exercise test was observed in the exercise training group but not the control group without exercise. Patients were trained in cycles of 2 min at 60–70% of the peak work rate in an incremental arm-crank test followed by 2 min of rest (20 min to 40 min session). One of the explanations for these changes could be the benefit of oxygen delivery to the untrained limbs due to an improvement in endothelial function resulting from aerobic exercise, an effect that was previously observed in [66]. The improvement in endothelial function is also favored by the anti-inflammatory effect of aerobic exercise. 

Currently, the use of home-based treatments significantly increases access to clinical care. Using a non-supervised, home-based exercise training program, Gardner et al. [47] observed significant increases in saturation level during exercise and at half time during resting recovery in the gastrocnemius, as occurred in the supervised exercise program (intermittent walking to mild-to-moderate claudication pain). This training consisted of 3 ses/wk for 3 months of intermittent walking with a progressive increase in pain claudication which were self-monitored with a step-counting monitor and the duration of which was to progressively increase from 20 to 45 min per session. This study demonstrated the benefits of exercise, including associated clinical improvements for patients with PAD, such as improved endothelial function and vascular anti-inflammatory effect, which are related to a decrease in the functional capacity of the individual, even at home when there is structure and control of exercise variables [67].

#### 3.4.2. Metabolic Muscle Diseases 

MMD are characterized by genetic mutations capable of altering lipid or glycogen metabolism or mitochondrial function (e.g., oxidative phosphorylation). This decrease in oxidative metabolism can lead to a decrease in the use of oxygen, reducing the demand imposed by the active musculature during exercise. A study conducted by Porcelli et al. [55] looked at the effects of 3 months of aerobic training (65–70% of HRmax), of 4 ses/wk at home, on a greater capacity to increase fractional O_2_ (∆[deoxy(Hb + Mb)]) extraction in vastus lateralis quadriceps and oxidative metabolism during low-intensity, constant exercise of patients with mitochondrial myopathy and McArdle’s disease. Here, both groups improved microvascular O_2_ delivery and uptake by the muscle (vastus lateralis quadriceps). These results were also associated with increased exercise tolerance and increased efficiency of oxidative metabolism after aerobic exercise training in MMD. 

#### 3.4.3. Chronic Kidney Disease 

In some cases, the musculature may be affected (i.e., myopathy) by chronic involvement of other organs, as occurs in end-stage renal disease (ESRD). The hyperparathyroidism, vitamin D deficiency, altered potassium metabolism, insulin resistance, uremic toxins, malnutrition, and physical deconditioning involved in this pathology favor muscle atrophy and weakness [68]. A study by Manfredini et al. [31] showed that 6 months of 4 ses/wk of 20 min of walking on non-dialysis days with progressive intensities starting at 70% of maximum walking speed up to 120% decreased the level of muscular oxygen consumption in the medial gastrocnemius at rest in patients with ESRD. An increased capacity of the oxidative metabolism (analyzing the rate of increase in HHb and ensuing venous occlusion) was related to this change [69,70]. However, a program of handgrip training for 4 days per week for 6 weeks at an initial intensity of 60% of the maximal voluntary contraction in the flexor digitorum superficialis muscle of CKD patients with hemodialysis with a progressively increasing number of repetitions each week showed no differences in vasodilator response measured by the ratio value of oxyhemoglobin after relief of arterial occlusion in relation to the control group [50]. This ratio corresponded to the recovery interval from occlusion release to the value obtained at baseline during rest. It is possible that the 6 weeks used by Kuge et al. [50] were too short to observe a good adaptation versus the 6 months used by Manfredini et al. [31]. On the other hand, the latter used aerobic exercise (walking) as opposed to the overload exercise (handgrip) used by Kuge et al. [50]. Previously, it was shown that lower volume and intensity are more conducive to vascular adaptation in these patients [71].

#### 3.4.4. Type 2 Diabetes Mellitus 

It is recognized that T2D produces a sustained increase in blood glucose levels, which favor a rise in endothelial oxidative damage, a lower affinity of hemoglobin for O_2_ (increased glycated hemoglobin) [72], and mitochondrial alteration to utilize O_2_ as an electron acceptor in complex 4 of the electron transport chain [73], affecting cellular aerobic metabolism. All these effects cause an alteration in peripheral vasodilator function (e.g., endothelial dysfunction), oxygen delivery, and metabolic inflexibility of the musculature, limiting tolerance to physical effort. A recent study by Gildea et al. [48] observed improved kinetics of muscle oxygen consumption (shorter phase 2 time) and a decrease in the ratio of deoxygenated hemoglobin plus muscle myoglobin in quadriceps (vastus lateralis) and lung oxygen consumption (∆[deoxy(HHb + Mb)]/VO_2_) in patients with T2D after 3 months of 3 ses/wk of MCT (50 min moderate-intensity cycling) and HIIT (10 reps of 1 min at 90% HRmax) [48]. Both parameters suggested an increase in the amount of microvascular blood to the active musculature and an improvement in oxygen delivery/utilization during exercise, respectively.

#### 3.4.5. Heart Failure

One disease that can affect oxygen delivery to the active muscle and thus alter energy production is heart failure with reduced ejection fraction (HFrEF) induced by acute myocardial infarction. The decrease in left ventricular contractile function leads to a decrease in the convective capacity of the cardiovascular system to promote blood distribution to the peripheral organs. Fu et al. [46] observed improvements in quadriceps muscle (vastus lateralis) oxygen extraction (Δ[HHb]) during exercise following a 3-month training program with interval aerobic exercise (five cycling intervals of 3 min at 80% VO_2_peak interspersed with five intervals of 3 min at 30% VO_2_max) in HFrEF patients but not following continuous, moderate exercise (30 min at 60% VO_2_peak) [46]. However, a study by Mezzani et al. [52] the same year observed a faster VO_2_ on-kinetics and an increase of peak skeletal muscle oxygen extraction measured with changes in deoxyhemoglobin during exercise in the vastus lateralis quadriceps of HFrEF patients after a 3-month training program with moderate exercise at ventilatory threshold one (five sessions of 30 min of cycling exercise for a week) [52]. The differences between the results of the two studies may be explained by variations in the volume of continuous moderate-intensity exercise training, as Fu et al. [46] performed fewer exercise sessions per week (3 ses/wk) than Mezzani et al. [52] (5 ses/wk) during the same 12 weeks of intervention. Most studies in patients with HFrEF recommend performing 5 to 6 ses/wk to achieve the desired effects [74,75]. A relevant aspect was the lack of oxygenation adaptation following MCT detected by NIRS in Fu et al.’s study [46], which agreed with a lack of peripheral vascular adaptation (Δ[tHb). Recently, Guimaraes et al. [49] observed that 30 min of continuous cycle ergometer exercise and one set of 10–15 reps (intensity 13–15 Borg scale) in five different resistance exercises improved exercise tolerance, muscle blood flow, and the ability to increase fractional O_2_ extraction through deoxyhemoglobin of the vastus lateralis quadriceps of HFrEF patients during the peak of a cardiopulmonary exercise test on a cycle ergometer: results dissimilar to Fu et al. [46], despite having used the same training volume (3 ses/wk for 12 weeks). It is possible that the higher intensity used in the study of Guimaraes et al. [49] compared to Fu et al. [46] may have allowed greater muscular adaptations, since, in the former, the subjects were trained at the respiratory compensation point, i.e., at ventilatory threshold two. The latter reached an intensity between 15 and 25% higher than ventilatory threshold one.

#### 3.4.6. Acute Myocardial Infarction

Coronary patients who have suffered an AMI due to ischemic thromboembolism caused by atheromatous plaque present, among other things, impaired myocardial function, increased arterial stiffness, increased blood pressure, and altered endothelial function. This limits the ability of blood flow redistribution to the active musculature and, therefore, the delivery of metabolic macromolecules (fatty acids and glucose) and oxygen to the muscle cells necessary for energy production. Takagi et al. [57], using 2 ses/wk of continuous aerobic exercise of mild-to-moderate intensity (10 watts below the lactate threshold) for 3 months, significantly increased exercise-induced desaturation measured with SmO_2_ in the vastus lateralis quadriceps in AMI patients during submaximal and maximal intensity during a cardiopulmonary exercise test. Even these results were associated with an increase in pulmonary VO_2_max, demonstrating improvements in the convective effect on oxygen (cardiac output) and its extraction by muscle in this type of patient.

#### 3.4.7. Orthopedic Disorders 

It is recognized that muscle disuse, as occurs after orthopedic surgery, causes a decrease in neuromuscular and/or metabolic capacity to increase physical exertion during contraction, affecting, among other things, oxygen consumption and redistribution of cardiac output in the area. A study by Olivier et al. [54] observed an increase in muscle oxygenation and blood flow in the quadriceps (vastus lateralis) of patients with ACLR after 6 weeks during a single-leg maximal exercise test versus in the control group (3 ses/wk of 21 min) of single-leg interval cycling (3 min at 70% and 3 min at 85% of peak heart rate). For that, muscle oxygenation was obtained by the relative changes in the oxy/deoxyhemoglobin/myoglobin concentrations and blood flow by changes in total hemoglobin, as was previously described [9,76,77]. During the intervention period, both groups also received a standard post-operative rehabilitation program. This consisted of therapy to decrease pain and swelling and to increase the range of motion of the knee in the first phase and then, in a second phase, the emphasis was on increasing the range of motion, increasing weight bearing, and gaining hamstring and quadriceps control [54]. However, in pathological contexts, such as in subjects with trapezius CM, a 10-week (3 ses/wk) program of aerobic training (20 min at 50–70% of VO_2_max), but not strength training (8–12 rep at 70–80% maximal resistance), showed greater blood flow by increased O_2_Hb and tHb during pegboard exercise [56]. Thus, the use of NIRS allowed us to observe the local muscular benefits of aerobic physical exercise on a healthy muscle in disuse or CM.

#### 3.4.8. Multiple Sclerosis

A lower O_2_ utilization capacity due to the damage produced on mitochondria in MS has previously been observed [78]. Using a pre–post study, Willingham et al. [79] showed an increase in resting muscle oxidative capacity analyzed with NIRS after 8 weeks of treadmill exercise in patients with MS and moderate-to-severe disability. Oxidative muscle capacity was analyzed by saturation during recovery after ischemic cuff occlusion by those such as Ryan et al. [80]. However, a clinical trial conducted by Manfredini et al. [51] looked at the effects of two sessions of 40 min of exercise per week for 1.5 months with either robot-assisted or physiotherapist-assisted exercise with pauses and the walking speed set according to the patient’s tolerance. Although both groups increased distance in the gait test and mitochondrial biomarkers, only the robot-assisted group improved resting oxidative capacity in the medial gastrocnemius (with muscle O_2_ diminished).

In terms of strengths, this review presents an exhaustive literature search; it focused only on clinical trials of muscle oxygenation, the eligibility criteria were explicit, a method for evaluating the quality of the articles specific to clinical trials was used, and pathologies that present central (heart failure) and peripheral (peripheral arterial disease, type 2 diabetes) aspects that could interfere with the results found were addressed. In addition, all the variables of the intervention protocols were highlighted, which was useful for the analysis of the results with the purpose of establishing incipient clinical evidence. The limitations of the review are that most of the clinical trials did not pass the acceptable quality limit, and there was a low number of studies per pathology, so extrapolating the results could be complex. One possible limitation of the search process was that it did not resort to the gray literature presented in clinical trial pages. (e.g., *ClinicalTrial.gov, WHO International Clinical Trials Registry Platform*, among others). Another relevant limitation is the lack of a meta-analysis since a review with meta-analysis can provide guidance in the design of future clinical trials.

However, to have more reliable results and/or conclusions, researchers should consider certain considerations/recommendations for the use of NIRS. One factor attenuating the passage of NIRS light into the muscles is subcutaneous adipose tissue. A significant increase in subcutaneous adipose tissue can significantly alter the estimation of NIRS variables. For the results to be comparable between different study populations, it is recommended when performing studies to select subjects with a homogeneous subcutaneous adipose tissue thickness in the NIRS reading area, ideally as low as possible, i.e., 20 mm, but never exceeding this [18]. It is also necessary to consider that significant heterogeneity of blood flow and muscle oxygen consumption in individuals has been previously identified. While healthy subjects can meet regional oxygen demands, those with clinical conditions, such as those seen in this review, may present greater heterogeneity (e.g., convective impairment of O_2_ delivery in HF or alterations of muscle metabolism in T2D), limiting oxygen flow between blood and myocytes. This is translated into a reduced delivery or utilization fractional measurement of muscle oxygen [81]. Finally, current NIRS equipment only allows the study of a small volume of muscle (superficially), the average depth of which only reaches half the distance between the light source and the receptor (i.e., ~1.5–3 cm approximately), which is not representative of what occurs in the rest of the muscle. It should be noted that oxidative muscle fibers are heterogeneously distributed among the different muscle groups [82]. Therefore, these aspects will have to be clarified in new studies to draw future general conclusions about the role of muscle oximetry in exercise science applied to health.

This review allowed the advantages and disadvantages of the use of NIRS in the evaluation of muscle oxygenation and peripheral blood flow adaptations to be clarified after clinical interventions in subjects with central and peripheral vascular risk factors, as well as cardiovascular, metabolic, and musculoskeletal alterations. This can guide therapeutic exercise specialists in making decisions regarding the prescription and monitoring of physical exercise as a therapeutic tool when observing either acute (actual treatment time) or chronic adaptations to improve efficiency in the treatment and recovery of these patients.

An important point is that NIRS, together with symptomatology, allows variables such as the type, intensity, recovery, and duration of exercise to be optimized, as well as the safety of its execution during therapy. However, more studies describing the physiological aspects involved in these adaptations are needed. Along with this, the quality of these future studies should be improved through strategies that favor the blinding of treatment providers, investigators, and patients.

## 4. Conclusions

In conclusion, NIRS proves to be a useful tool in observing muscle adaptive effects in randomized clinical trials using interval or continuous aerobic physical exercise and strength training and, therefore, can be used by exercise rehabilitation experts to guide recovery processes.

## Figures and Tables

**Table 1:** Quality scores of the PEDro scale applied to exercise clinical trials.

Studies	PEDro Quality Criteria												
	Selection				Comparability			Results				Rating	Quality
	1	2	3	4	5	6	7	8	9	10	11		
Baker et al., 2017 [44]	Yes	Yes	No	Yes	No	No	No	Yes	Yes	Yes	Yes	6	Medium
Beckitt et al., 2012 [43]	Yes	No	No	Yes	No	No	No	Yes	Yes	No	Yes	4	Medium
Collins et al., 2012 [45]	Yes	Yes	Yes	Yes	No	No	No	No	Yes	Yes	Yes	6	Medium
Fu et al., 2013 [46]	Yes	Yes	No	Yes	No	No	No	Yes	Yes	Yes	Yes	6	Medium
Gardner et al., 2014 [47]	Yes	Yes	Yes	Yes	No	No	No	Yes	Yes	Yes	Yes	7	Medium
Gildea et al., 2021 [48]	Yes	Yes	Yes	Yes	No	No	No	Yes	Yes	Yes	Yes	7	Medium
Guimarães et al., 2021 [49]	Yes	Yes	No	Yes	No	No	No	Yes	Yes	Yes	Yes	6	Medium
Kuge et al., 2005 [50]	Yes	No	No	Yes	No	No	No	Yes	Yes	Yes	Yes	5	Medium
Manfredini et al., 2012 [30]	Yes	No	No	Yes	No	No	No	Yes	Yes	Yes	Yes	5	Medium
Manfredini et al., 2015 [31]	Yes	Yes	No	Yes	No	No	No	Yes	Yes	Yes	Yes	6	Medium
Manfredini et al., 2020 [51]	Yes	Yes	No	Yes	No	No	No	Yes	Yes	Yes	Yes	6	Medium
Mezzani et al., 2013 [52]	Yes	Yes	No	Yes	No	No	Yes	Yes	Yes	Yes	Yes	7	Medium
Monteiro et al., 2019 [53]	Yes	Yes	No	Yes	No	No	Yes	No	Yes	Yes	Yes	6	Medium
Olivier et al., 2010 [54]	Yes	Yes	No	Yes	No	No	No	Yes	Yes	Yes	Yes	6	Medium
Porcelli et al., 2016 [55]	Yes	No	No	Yes	No	No	No	Yes	Yes	Yes	Yes	5	Medium
Søgaard et al., 2012 [56]	Yes	Yes	No	Yes	No	No	No	Yes	Yes	No	Yes	5	Medium
Takagi et al., 2016 [57]	Yes	No	No	Yes	No	No	No	Yes	Yes	Yes	Yes	5	Medium
Tew et al., 2009 [58]	Yes	Yes	Yes	Yes	No	No	No	Yes	Yes	Yes	Yes	7	Medium

**Table 2:** Characteristics of NIRS devices used in exercise clinical trials.

NIRS Device (Trademark, Model)	Technique	Measurements (Units)	Wavelength (nm)	Research Articles (Reference)
Artinis Medical Systems				
Portamon	CW, multi-distance	TSI (%), ∆HHb, ∆O_2_Hb, ∆tHb. (μmol)	750, 760, 841, 850	[46,53,55]
Oxymon Mk-III	CW, multi-distance	TSI (%), ∆HHb, ∆O_2_Hb, ∆tHb (μmol)	765, 770, 850, 905	[30,31,51]
Astem Co				
NIR srs Hb11	CW, multi-distance	StO_2_ (%)_,_ ∆HHb, ∆O_2_Hb, ∆tHb (μmol)	770, 830	[57]
Hamamatsu Photonics K.K.				
NIRO-300	CW, multi-distance	TOI (%), ∆HHb, ∆O_2_Hb, ∆tHb (μmol)	776, 826, 845, 905	[43,56,58]
NIRO-200	CW, multi-distance	TOI (%), ∆HHb, ∆O_2_Hb, ∆tHb (μmol)	735, 810, 850	[48]
Hutchinson Technology Inc.				
Inspectra Spectrometer 325	CW, multi-distance	StO_2_ (%)	680, 720, 760, 800	[45,47]
NIM Inc.				
	CW, multi-distance	StO_2_ (%), ∆HHb, ∆O_2_Hb, ∆tHb (arbitrary units)	730, 850	[54]
OMEGA				
BOM-L1TR	CW, multi-distance	StO_2_ (%), ∆HHb, ∆O_2_Hb, ∆tHb (μmol)	730, 810, 830	[50]
OMRON				
HEO-100	CW, single-distance	∆HHb, ∆O_2_Hb, ∆tHb (%)	760, 840	[52]
Thorlabs				
DCS FD-NIRS	FD, multi-distance	StO_2_ (%), aHHb, aO_2_Hb, atHb (μmol)	685, 785, 830	[44]
SS Inc., Champaign, IL				
Oxiplex TS	FD, multi-distance	SO_2_m (%), aHHb, aO_2_Hb, atHb (μmol)	692, 834	[49]

∆: relative changes; a: absolute changes; CW: continuous wave; FD: frequency domain; HHb: deoxyhemoglobin; O_2_Hb: oxyhemoglobin; tHb: total hemoglobin; SO_2_m/StO_2_/TOI/TSI: muscle oxyhemoglobin saturation.

**Table 3:** Clinical trials with exercise training that analyzed oxygenation, blood flow, and muscle metabolism with NIRS.

Author	Oxygenation Objective	Participants	Training Protocols	Intervention Length	Sampling Area	Results after Training Protocol
Baker et al., 2017 [44]	AET on microvascular blood flow and muscle oxygen extraction in PAD	64 pt. with PAD. AET (*n* = 29): 66 (59, 69) y. CON (*n* = 35): 67 (60, 76) y	AET: 60 min of treadmill walking intervals at 2 mph with increasing gradient (2%/2 min) until claudication. CON: non-exercise	3 ses/wk/ 3 mo	Gastrocnemius	AET: Higher blood flow and oxygen desaturation during maximal exercise test. CON: without changes
Beckitt et al., 2012 [43]	Exercise training (ET) versus angioplasty (AG) on oxygen muscle saturation in stable claudication patients	56 pt. with stable claudication PAD. ET (*n* = 42): 66 ± 6.1 y. AG (*n* = 14): 68 ± 5.8 y	ET: 10′ warm-up, 5 station circuit, 8′ each station. AG: angioplasty without exercise	2 ses/wk/ 3 mo	Lateral gastrocnemius	ET and AG: Higher reoxygenation during recovery after an ischemia occlusion AG: A lower hemoglobin desaturation during submaximal exercise test
Collins et al., 2012 [45]	Oxygen muscle saturation in PAD after a TWP versus a WPP exercise program in PAD	85 pt. with PAD. TWP (*n* = 40): 66.8 ± 8.5 y. WPP (*n* = 45): 71.7 ± 9.2 y	TWP and WPP: 9 wk. for 6 min at 25–44% VO_2_peak (LIn), 18 min at 45–59% VO_2_peak (MIn), and 6 min at 60–84% VO_2_peak (HI) according to maximal pain tolerance. Following ~3 wk, pt. walked for 5.5 min at LI, ~25 min at MI, and ~25 min at HI (WPP: with poles)	3 ses/wk/ 3 mo	Medial gastrocnemius	TWP: Higher muscle saturation during submaximal intensity in a treadmill exercise test. WWP: without changes
Fu et al., 2013 [46]	MCT and AIT on central and peripheral hemodynamics in heart failure (HF)	45 pt. with HF. AIT (*n* = 15): 67.5 ± 1.8 y. MCT (*n* = 15): 66.3 ± 2.1 y. CON (*n* = 15): 67.8 ± 2.5 y	AIT: Five cycling intervals of 3 min at 80%, VO_2_peak interspersed with 5 intervals of 3 min at 30% VO_2_max. MCT: 30 min at 60% VO_2_peak. CON: non-exercise	3 ses/wk/ 3 mo	Vastus lateralis quadriceps	AIT: Higher oxygen extraction muscle during all exercise in a maximal test. CON: without changes
Gardner et al., 2014 [47]	Muscle oxygenation in PAD after supervised exercise training (SET), home-exercise program (HEP), or an attention control group (CON)	180 pt. with PAD. SET (*n* = 60) 65 ± 11 y. HEP (*n* = 60) 67 ± 10 y. CON (*n* = 60) 65 ± 9 y	SET: ITW to mild-to-moderate claudication pain at a speed of 2 mph at 40% maximal power output in maximal treadmill test with increase from 15 to 40 min. HEP: ITW to mild-to-moderate claudication pain at a self-selected pace with a step monitor, increasing 20 to 45 min per session. CON: non-exercise	3 ses/wk/ 3 mo	Gastrocnemius	SET and HEP: Higher saturation level during submaximal intensity exercise and at half time during resting recovery. CON: without changes
Gildea et al., 2021 [48]	Muscle VO_2_ and oxygenation kinetics after HIIT and MCT in T2D	28 pt. with T2D. MCT (*n* = 10): 53 ± 10 y. HIIT (*n* = 9): 52 ± 10 y. CON (*n* = 9): 54 ± 9 y	MCT: 50 min moderate-intensity cycling. HIIT: 10 reps of 1 min at 90% HRmax. CON: non-exercise	3 ses/wk/ 3 mo	Vastus lateralis quadriceps	HIIT and MCT: Improved the VO_2_ kinetics (↓ tau) and decreased muscle deoxygenation (↓ ∆[HHb + Mb]/dVO_2_) during exercise. CON: without changes
Guimaraes et al., 2021 [49]	AET plus resistance training (ART) on peripheral muscular performance and muscle oxygenation in HF	24 pt. with HF. HF-ART (*n* = 16): 49 ± 9 y. HF-CON (*n* = 8): 46 ± 5.8 y	HF-ART: 30 min of AET on cycle ergometer at CRP and 1 set of 10–15 reps (intensity 13–15 Borg scale) in 5 different resistance exercises. HF-CON: non-exercise	3 ses/wk/ 3 mo	Vastus lateralis quadriceps	HF-ART: Higher muscle oxygenation (↓Oxy-Hb, ↓ deoxy-Hb, and ↓ tHB) during peak in an exercise test. HF-CON: without changes
Kuge et al., 2005 [50]	Vasodilator response, muscle oxygenation, and performance post exercise in hemodialysis patients (HP) by CKD	15 subjects. HP (*n* = 8): 61.1 ± 5.8 y. CON (*n* = 7; healthy subjects): 58.7 ± 5.8 y	HP: Handgrip training for 4 d/wk during 6 wk (15 to 30 min app.). 50 reps at 60% MVC during 1st wk, increasing 20 reps/wk until reaching 150 reps. CON: non-exercise	4 ses/wk/ 1.5 mo	Flexor digitorum superficialis	HP: Without changes in vasodilator response (↔[tHb]) but a higher muscle reoxygenation (StO_2_) after 3 min arterial occlusion. CON: without changes
Manfredini et al., 2012 [30]	Structured (SW) versus unstructured walking (UW) program exercise on hemodynamic, functional, and muscle VO_2_	45 pt. with PAD. SW (*n* = 31): 71.9 ± 6.4 y. UW: 70.3 ± 7.4 (*n* = 14) y. CON: (*n* = 15, healthy subjects): 38.3 ± 15.3 y	SW: 2 rep/d of 10 min of walking at 20–30% below pain threshold speed. UW: free walking 20 to 30 min/d to a moderate level of pain. CON: non-exercise	6 ses/wk/ 8.5 mo	Medial gastrocnemius	SW: Increased the mVO_2_ (↑ the rate of increase in (HHb) during venous occlusion) to healthy subject values and perfusion (ABI) at rest, indicating normalized muscle function. Increased the distance to claudication during exercise. UW: without changes
Manfredini et al., 2015 [31]	Walking exercise on resting mVO_2_ and vascular function in myopathy for end-stage renal disease (ESRD)	54 pt. myopathy by ESRD. EXP (*n* = 28): 66 ± 14 y. CON (*n* = 26): 68 ± 13 y	EXP: 2 rep/d of 10 min of walking at 70–120% of maximum walking speed. CON: recommendations for maintaining an active lifestyle	4 ses/wk/ 6 mo	Medial gastrocnemius	EXP: Decreased the mVO_2_ (idem [30]) at rest, indicating lower muscle dysfunction. CON: without changes
Manfredini et al., 2020 [51]	Robot (RO)- and physiotherapist (PT)- assisted walking on mVO_2_ in multiple sclerosis (MS)	46 pt. with MS and 10 control healthy subjects. MS-RO (*n* = 23). MS-PT (*n* = 23). CON (*n* = 10)	MS-RO: 40 min of robot-assisted walking. MS-PT: 40 min of walking assisted by physiotherapists. CON: non-exercise	2 ses/wk/ 1.5 mo	Medial gastrocnemius	MS-RO: Decreased the mVO_2_ rest (idem [30]). MS-PT and CON: without changes
Mezzani et al., 2013 [52]	AET effects on pulmonary and muscle VO_2_ kinetics in heart failure	30 pt. with HF and 7 healthy subjects. HF-AET (*n* = 15): 65 ± 7 y. HF-CON (*n* = 15): 63 ± 7 y. CON (*n* = 7): 66 ± 4 y	HF-EXP: 30 min cycling exercise at ventilatory threshold 1. HF-CON: habitual lifestyle and activities without a formal training protocol. H-CON: non-exercise	5 ses/wk/ 3 mo	Vastus lateralis quadriceps	HF-EXP: Decreased pulmonary time delay of VO_2_ kinetics during submaximal steady-state exercise and increased peak oxygen extraction in muscle during maximal exercise test (↑ peak ∆[HHB]). HF-CON: without changes
Monteiro et al., 2019 [53]	Muscle oxygenation in PAD after MCT versus modified aerobic training (MAT) with a load on the lower limbs	40 pt. with PADMCT (*n* = 20): 65.4 ± 10.6 y. MAT (*n* = 20) 63.1 ± 10.5 y	MCT: 30 min of walking on the floor and 30 min on treadmill at floor walking speed without inclination (increase of 0.2 km/h with the cessation of symptoms). MAT: 15 min of walking on the floor with ankle weights (increase progressively from 0.5 to 2 kg). Both trainings were symptoms controlled	3 ses/wk/ 3 mo	Medial gastrocnemius	MCT and MAT: Decrease the rate of muscle desaturation (StO_2_) with MCT > MAT. MCT: Higher muscle saturation during maximal exercise test (↑ in exercise test duration)
Olivier et al., 2010 [54]	One leg cycling training on leg muscle oxygenation (LMO_2_) in soccer players with anterior cruciate ligament reconstruction	24 regional-level soccer players with ACLR. EXP (*n* = 12): 25.1 ± 3.4 y. CON (*n* = 12): 23.2 ± 3.1 y	EXP: 21 min alternating 3 min at 70% HRmax and 3 min at 85% HRmax. CON: familiarization training during 10 min at 30 W	3 ses (CON: 1 ses)/wk/ 1.5 mo	Vastus lateralis quadriceps	EXP: Increased in LMO_2_ (relative change in the oxy/deoxy hemoglobin/myoglobin) and blood flow (changes in tHB) during one leg maximal graded test. CON: without changes
Porcelli et al., 2016 [55]	Home-based AET on muscle oxygen uptake and fractional O_2_ extraction in mitochondrial myopathies (MM) and McArdle’s disease (McA)	13 patients with mitochondrial myopathies. MM (*n* = 6): 51 ± 1.6 y. McA (*n* = 7): 41 ± 1.3 y	MM and McA: 30 min of cycling (wk 1–6) and 45 min (wk 7–12) at 65–70% of HRmax	4 ses/wk/ 3 mo	Vastus lateralis quadriceps	MM and McA: Higher changes in skeletal muscle fractional O_2_ extraction (deoxy(Hb + Mb)) during exercise
Søgaard et al., 2012 [56]	General fitness training (GFT) performed as leg bicycling versus strength training (ST) on muscle oxygenation in trapezius with chronic myalgia	39 pt. with trapezius myalgia. GAT (*n* = 15): 45.5 ± 8.0 y. ST (*n* = 16): 44.6 ± 8.5 y. CON (*n* = 8): 42.5 ± 11.1 y	GFT: 20′ at 50–70% of VO_2_max. ST: 8–12 rep at 70–80% MR. CON: non-exercise	3 ses/wk/ 2.5 mo	Trapezius muscle	GFT: Higher blood flow (↑ in O_2_Hb and tHB) during pegboard exercise. CON: without changes
Takagi et al., 2016 [57]	AET on muscle deoxygenation and VO_2_peak in post-myocardial infarction (AMI)	16 pt. with AMI. AET (*n* = 10): 59 ± 10 y. CON (*n* = 6): 61 ± 9 y	AET: 10 W below LT, 30’ x 10′ warm up and 10′ cool down. CON: non-exercise	2 ses/wk/ 3 mo	Vastus lateralis quadriceps	AET: Higher muscle oxigenation (↓ SmO_2_ and ↑ deoxy-Hb) during submaximal and peak intensity in a maximal exercise test. CON: without changes
Tew et al., 2009 [58]	Arm-crank exercise (ACE) training on lower-limb O_2_ delivery in patients with intermittent claudication	57 pt. with PAD. ACE (*n* = 27): 69 ± 9 y. CON (*n* = 24): 70 ± 8 y	ACE: Cycles of 2 min exercise at a crank rate of 50 rev/min at 60–70% of the peak work rate in an incremental arm-crank test followed by 2 min of rest for a total exercise time of 20 min in a 40 min session. CON: non-exercise	2 ses/wk/ 3 mo	Gastrocnemius	ACE: Higher submaximal oxygenation (↑ StO_2_) during maximal exercise testing. CON: without changes

ABI: Ankle-brachial index; AET: Aerobic exercise training; AIT: Aerobic interval training; CKD: Chronic kidney disease; CON: Control group; EXP: Experimental group; HI: High intensity; HIIT: High-intensity interval training; ITW: Intermittent treadmill walking; LIn: Light intensity; MIn: Moderate intensity; MCT: Moderate continuous training; MVC: Maximal voluntary contraction; mVO_2_: Muscular oxygen consumption; PAD: Peripheral arterial disease; RCP: Respiratory compensation point; T2D: Type 2 diabetes mellitus: TWP: Traditional walking program; WPP: Walking-with-poles program. ↑ or ↓: significant changes; ↔: no significant changes.

## Data Availability

Not applicable.

## Appendix A

**Table A1:** Search strategy in clinical studies.

Steps	Strategy	PubMed (444)	WoS (47)	Scopus (302)
1	Spectroscopy, Near-Infrared	1101	473	1621
2	NIRS	417	150	558
3	Exercise	65,369	21,026	81,580
4	Exercise therapy	33,931	1299	14,021
5	Physical exertion	5735	58	1050
6	Physical Fitness	6696	543	3968
7	Sports	34,562	2502	9176
8	Exercise Movement Techniques	2820	24	321
9	#3 OR #4 OR #5 OR #6 OR #7 OR #8	74,620	24,939	87,414
10	#1 AND #9	343	37	246
11	#2 AND #9	101	10	83
